# Measuring drinking motives in undergraduates: an exploration of the Drinking Motives Questionnaire-Revised in Swedish students

**DOI:** 10.1186/s13011-019-0239-9

**Published:** 2019-11-08

**Authors:** Christina Nehlin, Caisa Öster

**Affiliations:** 1 Department of Neuroscience, Psychiatry, Uppsala University, UAS entr 10, SE-751 85 Uppsala, Sweden; 2 Uppsala University Hospital, UAS entr 10, SE-75185 Uppsala, Sweden

**Keywords:** Drinking motives, Students, Questionnaires, Validation

## Abstract

**Background:**

Alcohol consumption is generally high among undergraduate students and may lead to adverse consequences. Drinking motives play a vital role in the development of alcohol-related problems. The Drinking Motives Questionnaire-Revised (DMQ-R) and the short form of DMQ-R, DMQ-R SF, are widely used tools to identify drinking motives. Still, there is a need for further exploration of the instruments in different cultures and settings. The aims of this study were 1) to explore the four-factor structure of the DMQ-R and DMQ-R SF in Swedish undergraduate students 2) to investigate if extracting the SF responses from the DMQ-R is equivalent to the factor structure of the DMQ-R SF 3) to study the association between drinking motives and hazardous drinking.

**Methods:**

Data were collected among 536 Swedish undergraduate students and were analyzed by confirmatory factor analyses, Mann-Whitney, chi-square tests and logistic regressions.

**Results:**

We could confirm the four-dimensional structure of both versions of the DMQ. There was a similar (or in fact even slightly better) model fit of the short form and when drawing the SF items. Emotionally oriented motives (enhancement and coping), together with social motives, were strongly associated with hazardous or harmful drinking levels, whereas conformity motives were not. The enhancement motive showed the highest group mean value and was also the most common main motive. Students with hazardous drinking endorsed their motives more strongly than those without hazardous drinking, which is a finding worthy of further investigation.

**Conclusions:**

The DMQ-R SF is suitable and preferable for Swedish student populations and extracting the SF responses from the DMQ-R is equivalent to the factor structure of the DMQ-R SF. In future research, effects of including the DMQ-R SF in preventive strategies and in interventions with risk drinking students would be of particular interest.

## Background

The university years do not only involve personal growth and intellectual development. They are also a period in which many students consume large quantities of alcohol and experience a number of associated adverse effects [1]. Because a large proportion of young people enter a university program during their lifetime, the university is an important arena for the prevention of future alcohol problems as well as for targeted interventions.

Research suggests that drinking motives are the most proximal predictors of the quantity and frequency of alcohol use among young people [2, 3]. Furthermore, associations between specific drinking motives and drinking patterns have been traced. Social motives, the most commonly reported motives in young people, are linked to moderate drinking levels, whereas enhancement or coping motives are related to heavy drinking [2]. It is suggested that drinking motives in young age may predict drinking patterns later in life [4]. Preliminary evidence also indicates that targeting drinking motives may be useful in reducing problematic drinking [5].

Measuring drinking motives in a consistent and reliable manner is therefore of importance.

The Drinking Motives Questionnaire (DMQ), originally developed for college student populations, is the most widely used measure of drinking motives [6, 7]. In its first version, the questionnaire comprised 15 items, grouped under three motivational components: enhancement (to improve positive mood state), coping (to relieve negative mood state) and social (to obtain social benefits). In 1994 a fourth motive, conformity (to avoid social rejection), was added to the DMQ. This version, the DMQ-Revised (DMQ-R), contains 20 items [8]. A short form that contains 12 items from the original DMQ-R, the DMQ-R SF (Short Form), has been developed and validated in Swiss and Italian adolescents [9, 10] as well as in an American sample of underage drinkers [11]. A recent study including 10 countries found good psychometric properties of the SF across different cultural settings [12]. Both variants measure four motives: enhancement, coping, conformity and social. In some studies, the SF items are drawn from the full DMQ-R instead of using the SF. It may be considered methodologically problematic to draw short-form items from the responses of a more extensive questionnaire (e.g. [11]).

## Aims

To our knowledge, no studies have been reported comparing the DMQ-R and DMQ-R SF in university settings, nor have the questionnaires been tested in a non-clinical Swedish setting. The present study, therefore, aimed to 1) explore the four-factor structure of DMQ-R SF in Swedish undergraduate students and to compare the model fit with DMQ-R 2) to investigate if extracting the SF responses from the DMQ-R is equivalent to the factor structure of the DMQ-R SF and 3) to study the association between drinking motives and hazardous drinking.

## Methods

Data were collected between September 2016 and August 2017 from a diverse convenience sample of undergraduate students enrolled in courses at Uppsala University, Sweden (the courses included social work, nursing, veterinary medicine, psychology, medicine or introductory natural science). Data were collected in two separate student samples: one using the DMQ-R and another using the DMQ-R SF. A research assistant presented the project to the students during a regular lecture class. Participation was voluntary, and confidentiality was assured. Those who accepted to participate responded anonymously during a break or after the lecture. Respondents took about 10 min to complete the questionnaire.

### Measures

The English version of the DMQ-R was translated into Swedish. The DMQ-R consists of 20 items and the SF of 12 items [8, 9]. Participants rated frequency of drinking for each item on an ordinal scale with six response categories (1 = never, 2 = almost never, 3 = some of the time, 4 = about half of the time, 5 = most of the time, 6 = almost always).

The AUDIT (Alcohol Use Disorders Identification Test) [13] is a 10-item screening instrument designed to identify hazardous alcohol habits. Each item is scored on a scale from 0 to 4. Internal consistencies for AUDIT sum score were satisfactory (Cronbach’s alpha 0.74). In this study AUDIT was only used to distinguish persons with hazardous from those with non-hazardous drinking. We used the recommended separate cut-off levels for women (6 points) and men (8 points) [14].

### Data analysis

To confirm the factor structure of the DMQ-R SF a confirmatory factor analysis (CFA) was run, with parallel analyses using 1) the DMQ-R SF 2) the original DMQ-R and in addition 3) DMQ-R SF as a subset of items from the DMQ-R. Parameters in the CFA were estimated using the maximum-likelihood procedure. The model fit indices used were the CFI (comparative fit index), SRMR (standardized root mean square residual) and RMSEA (root mean square error of approximation). A CFI higher than 0.90 (preferably close to 0.95), a SRMR close to 0.08 or smaller and a RMSEA close to 0.07 or smaller indicate a good fit [15].

Differences between subgroups were analysed with the Mann-Whitney and chi-square tests. Principal drinking motive for a participant was identified as the motive with the highest mean subscale score.

To investigate associations between AUDIT scores indicating hazardous drinking and independent variables we performed a logistic regression. All independent variables was entered simultaneously into the equation in one step: A score of 6 for females and 8 for males on the AUDIT served as the dependent variable (non- hazardous drinking = 0, hazardous drinking = 1). Variables were the four motives from the DMQ-R SF together with age and sex (male = 0, female = 1). Correlations between the variables were 0.02 (coping and social) to 0.65. (enhancement and social). Nagelkerke *R*^2^ was used to estimate the proportion of variance explained. Statistical significance was set at *p* < 0.05. Statistical analyses were performed using the statistical packages IBM SPSS Statistics 21.0 and CFA was performed using the RStudio.

## Results

In all, 536 students who declared they had consumed alcohol in the past 12 months (AUDIT item #1 ≥ 1) were included. Of those, 211 (39.4%) had responded to the DMQ-R SF and 325 (60.6%) to the DMQ-R. The characteristics of the participants are listed in Table 1.

**Table 1:** Demographic characteristics of participants and subgroups

	Total	DMQ-R SF	DMQ-R	Non-hazardous drinkers	Hazardous drinkers^1^
	n (%)			n (%)	
Participants	536 (100)	211 (39.4)	325 (60.6)	227 (42.4)	309 (57.6)
Women	329 (61.6)	140 (66.4)	189 (58.2)	116 (35.3)	213 (64.7)
Men	207 (38.4)	71 (33.5)	136 (41.8)	111 (53.6)	96 (46.4)***
Risk drinkers ^1^	309 (57.3)	123 (58.0)	186 (57.1)		
Mean ± SD Md (range)					
Age ± SD	22.0 ± 2.7 21.0 (17–30)	21.8 ± 2.5 21.0 (18–30)	22.1 ± 2.9 22.0 (17–30)	22.1 ± 2.4 22.0 (17–30)	21.9 ± 3.0 21.0 (18–30)
AUDIT Sum	7.7 ± 4.5^2^ 7.0 (1–25)	7.6 ± 4.7 7.0 (1–25)	7.7 ± 4.3 7.0 (1–22)	3.9 ± 1.7 4.0 (1–7)	10.4 ± 3.8 10.0 (6–25)***
*Mean score DMQ*					
Enhancement	3.9 ± 1.4 4.0 (1–6)	3.8 ± 1.4 3.7 (1–6)	3.9 ± 1.3 4.0 (1–6)	3.1 ± 1.1 3.0 (1–6)	4.4 ± 1.4 4.7 (1–6)***
Social	3.7 ± 1.3 3.7 (1–6)	3.5 ± 1.4 3.7 (1–6)	3.8 ± 1.3 3.7 (1–6)*	3.1 ± 1.4 3.0 (1–6)	4.2 ± 1.1 4.3 (1–6)***
Coping	1.7 ± 0.9 1.3 (1–6)	1.7 ± 0.9 1.3 (1–6)	1.7 ± 1.0 1.3 (1–6)	1.4 ± 0.7 1.0 (1–6)	1.9 ± 1.0 1.7 (1–6)***
Conformity	1.7 ± 0.8 1.3 (1–5.7)	1.8 ± 0.8 1.7 (1–5)	1.6 ± 0.9* 1.3 (1–5.7)	1.7 ± 0.8 1.3 (1–5)	1.7 ± 0.9 1.3 (1–5.7)

1) AUDIT (Alcohol Use Disorders Identification Test) sum score ≥ 6/8 female/male. Mean AUDIT Sum score Women 6.8 ± 4.3, 6 (1–24) , Mean AUDIT Sum score Men 7.6 ± 5.1, 7(125). Chi-square and Mann-Whitney test, * *p* < .05, ***p < .001

In 79.2% of the students (*n* = 427) one single principal motive was identified. Enhancement was the most commonly expressed motive (45.9%, *n* = 246), followed by the social (28.9%, *n* = 155), conformity (4.1%, *n* = 22) and coping (0.7%, n = 4) motives.

### Confirmatory factor analyses

Fit statistics for the DMQ-R SF suggested that the model had a relatively good fit (RMSEA = 0.08, CFI = 0.96, SRMR = 0.05) [15] and the fit indices were highly similar to those in the original DMQ-R with 20 items (RMSEA = 0.09, CFI = 0.95, SRMR = 0.05). To further test the short form of the DMQ-R, analyses of DMQ-R SF as a subset of items from the DMQ-R were conducted and resulted in a comparable fit (RMSEA = 0.06, CFI = 0.98, SRMR = 0.05).

The results of the CFA revealed high and homogenous item loadings with 0.57 or higher, except for one item in the original DMQ-R “Because you feel more self-confident or sure of yourself?” (Table 2). In all three analyses the drinking motives factors were significantly correlated with the highest correlation between enhancement and social factors (original DMQ-R SF 0.89, DMQ-R 0.81, DMQ-R SF as a subset of DMQ-R 0.89). All four subscales in the three DMQ-R measures demonstrated reasonable internal consistency estimates of ≥0.83.

**Table 2:** Results of confirmatory factor analyses (standardized item loadings), interfactor correlations and internal consistencies

	DMQ-R				DMQ-R SF				DMQ-R SF*			
How often do you drink	En	So	Co	Cf	En	So	Co	CF	En	So	Co	Cf
*Enhancement*												
** 1. because you like the feeling?**	.82				.85				.79			
5. because it’s exciting?	.80											
** 9. to get high?**	.78				.82				.77			
** 13. because it gives you a pleasant feeling?**	.81											
** 17. because it’s fun?**	.85				.82				.88			
*Social*												
** 4. because it helps you enjoy a party?**		.88				.92				.88		
8. to be sociable?		.57										
**12. because it makes social gatherings more fun?**		.78				.79				.77		
** 16. because it improves parties and celebrations?**		.92				.91				.94		
20. to celebrate a special occasion with friends?		.59										
*Coping*												
2. to forget your worries?			.81									
** 6. because it helps you when you feel depressed or nervous?**			.89				.84				.92	
** 10. to cheer up when you’re in a bad mood?**			.80				.80				.82	
14. because you feel more self-confident or sure of yourself?			.42									
** 18. to forget about your problems?**			.92				.88				.88	
*Conformity*												
3. because your friends pressure you to drink?				.64								
7. so that others won’t kid you about not drinking?				.64								
** 11. … to fit in with a group you like?**				.81				.89				.84
** 15. to be liked?**				.74				.79				.79
** 19. so you won’t feel left out?**				.74				.91				.70
Correlations with the factor social	.69		.39	.13	.78		.45	.35	.67		.25	.12
Correlations with the factor coping	.43	.39		.23	.49	.45		.37	.32	.25		.18
Correlations with the factor conformity	.10	.13	.23		.23	.35	.37		.05	.12	.18	
Internal consistency (Cronbach’s alpha)	.89	.82	.85	.84	.84	.88	.88	.86	.83	.87	.91	.82

Factor loadings are standardized item loadings. Factor loadings and correlations are significant at *p* < .001. Enhancement = En, Social = So, Coping = Co, Conformity = Cf. Items in DMQ-R SF in bold. DMQ-R SF* = as a subset of items from the DMQ-R (the same sample who used the original DMQ-R)

### Hazardous drinking

In the model obtained with logistic regression (Table 3), being a woman together with three of the drinking motives (enhancement, social and coping) was related to hazardous drinking.

**Table 3:** Logistic regression with drinking behavior indicating hazardous alcohol use^*^ as the dependent variable.

Independent variables	Odds ratio	95% CI	*p* value
Age	1.1	1.0–1.1	.125
Sex	2.5	1.6–3.8	<.001
Enhancement	1.7	1.4–2.1	<.001
Social	1.4	1.1–1.7	.003
Coping	1.5	1.1–2.0	.004
Conformity	0.9	0.7–1.2	.390
Nagelkerke’s R Square = 0.34			

The four DMQ motives = mean values. Sex (Men = 0, Women =1). CI, Confidence interval

*) AUDIT (Alcohol Use Disorders Identification Test) sum score ≥ 6/8 female/male

## Discussion

As hypothesized, we could confirm the four-dimensional structure by using the DMQ-R SF in a population of Swedish undergraduate students. We also found, as expected, a similar (or in fact a slightly better) model fit of the short form in the Swedish student sample. We can therefore confirm previous findings in adolescent and clinical samples in Europe and in the USA [9, 11, 16]. However, our results do not correspond with the findings from a general population sample in China, [17]. In that study, a poorer overall model fit was found in the 12-item version DMQ-R SF than in the original 20-item version. Cultural and study procedural differences may explain the discrepancy between the studies.

In line with previous research [2], we found that emotionally oriented motives (enhancement and coping) were strongly associated with hazardous or harmful drinking levels together with social motives and that no significant association with those drinking levels was seen for conformity motives. One explanation could be that young people regulate their feelings with alcohol and use it to facilitate interaction [2, 18].

Enhancement was the motive with the highest group mean value and was also the most common principal motive. Accordingly, in previous studies from our group in adults in psychiatric treatment and in persons with drinking problems, enhancement has been the most commonly expressed motive [16, 19]. In other studies of drinking motives in high school and college students and in adults, social has been the most frequently indicated motive [11, 20, 21]. Cultural implications of what drinking is good for may have played a role in the students’ reported motives. How different drinking traditions may affect young persons’ dealing with alcohol has been discussed in previous studies (e.g. [7, 22]), but further research on this subject is warranted.

Similar to previous studies, we found a strong correlation between the enhancement and the social motive (e.g. [23]). The word “fun” (“roligt” in Swedish) is used in both an enhancement and a social item, which may be an explanation for higher correlations. However, when we run this regression as a stepwise model both enhancement and social motive are significantly included in the model. We therefore conclude that the results are not violated due to the correlation between enhancement and social motive.

A noteworthy finding is that the students drinking at hazardous or harmful levels endorsed their motives more strongly. Except for conformity motives, the mean values of motives were significantly higher in risk drinkers than in non-risk drinkers. This observation could be due to misinterpretation of the questionnaire: The question is not, “How often do you drink?” but rather “Out of all your drinking occasions, how many of these were you drinking because …? ” People who drink more often may tend to give higher values to each motive. If so, strongly endorsed motives may predict risk-drinking, which, in turn, may have implications for prevention strategies. This issue has not been investigated and other explanations for the stronger endorsement of motives are possible.

Limitations of the study need to be addressed. Self-reported data, especially on alcohol use, always carry the risk that participants do not adequately report the consumption. Social desirability can play a role in not being forthright in the report of alcohol use. The aim of this study, however, is to explore the structure of different versions of the DMQ-R. A potential underrating of drinking levels does not affect the comparison of the instruments.

## Conclusions

We conclude that the DMQ-R SF is suitable for Swedish student populations and that drawing the short form items from the original DMQ-R seems to perform as well as the DMQ-R SF itself. In line with previous findings, we found enhancement, social or coping motives to be related to hazardous drinking; but unlike other studies we found enhancement to be the most strongly endorsed motive. The endorsement of motives is stronger in hazardous drinkers than in non-hazardous drinkers. Reasons for this need further investigation.

Responding to alcohol-related questionnaires may have an impact on drinking habits [24]. So far, only a few studies have reported using motives as a treatment component [5, 25]. In future research, effects of including the DMQ-R SF in preventive strategies as well as in interventions with risk drinking students would be of particular interest.

## Abbreviations

AUDIT: The Alcohol Use Disorders Identification Test
CFA: Confirmatory Factor Analysis
CFI: Comparative Fit Index
DMQ: The Drinking Motives Questionnaire
DMQ-R: The Drinking Motives Questionnaire-Revised
DMQ-R SF: The Drinking Motives Questionnaire-Revised Short Form
RMSEA: Root Mean Square Error of Approximation
SF: Short Form
SRMR: Standardized Root Mean Square Residual
